# Have We Taken Advantage of the Quarantine to Develop Healthy Habits? A Cross-Sectional Analysis of the Spanish COVID-19 Situation by Gender

**DOI:** 10.3390/healthcare9070844

**Published:** 2021-07-04

**Authors:** Miguel Corbí, Mario Del Líbano, Almudena Alonso-Centeno, Aida Gutiérrez-García

**Affiliations:** 1 Departamento de Didácticas Específicas, Facultad de Educación, Universidad de Burgos, 09001 Burgos, Spain; mcorbi@ubu.es (M.C.); aacenteno@ubu.es (A.A.-C.); 2 Departamento de Ciencias de la Educación, Facultad de Educación, Universidad de Burgos, 09001 Burgos, Spain; 3 Departamento de Ciencias de la Salud, Facultad de Ciencias de la Salud, Universidad de Burgos, 09001 Burgos, Spain; aidagg@ubu.es

**Keywords:** social determinants, healthy habits, physical activity, diet, psychological well-being, COVID-19

## Abstract

The confinement caused by the COVID-19 pandemic led to changes in people’s lifestyles, which in part provided an opportunity to develop habits at home. The aims were: (1) to verify if the psychological well-being (PWB) of people related to healthy habits, and if physical activity (PA) and diet mediated this relationship; (2) to test if there were differences in this model of relationships between women and men; (3) to analyze if there were differences in healthy habits, PA, diet, and PWB depending on gender; (4) to test if there were differences in healthy habits, PA, diet, and PWB depending on living area; (5) and to assess if there were interaction effects of gender and living area in healthy habits, PA, diet, and PWB. Using a cross-sectional design, we obtained a sample of 1509 participants (18–78 years, 1020 women). Diet and PA fully mediated the relationship between PWB and healthy habits, and women developed more healthy habits than men, whereas men had higher levels of PA and PWB. We also found that people who lived in rural areas during confinement practiced more PA and had lower PWB levels than those who lived in urban areas. These results can help in the planning of strategies to promote healthy habits.

## 1. Introduction

The promotion of health remains a crucial challenge for governments globally who need to find innovative and effective approaches [1] within and outside the health care system. In recent decades, the term “social determinants of health” has emerged as a foundational concept in the field of population and public health [2]. Although there are some ambiguities and confusion about this term [3], we can define it as the conditions in which people develop their lives, including factors like socioeconomic status, education, neighborhood and physical environment, employment, social support networks, and access to health care [4,5]. These factors may create differences among population groups that underlie health conditions, making them more vulnerable to consequences [6] and resulting in diverse changes in their lifestyle. Therefore, social determinants may affect behavioral aspects that lead to health outcomes [7,8,9].

In 2020, the world experienced such an exceptional circumstance that the usual social determinants were affected at all levels. The spread of the virus called SARS-CoV2 (Severe Acute Respiratory Syndrome Coronavirus 2), which gave rise to the disease known as COVID-19 (COronaVIrus Disease 2019), led the World Health Organization (WHO) to declare a global pandemic in March 2020 [10]. As a result, the governments of many countries were forced to make difficult decisions, such as enforcing restrictions to contain the diffusion of the virus, extending as far as the exceptional measure of quarantine; in contrast, other countries recommended populations remain isolated as much as possible. Both isolation and quarantine showed good results in the past in the restoration of public health [11], although these measures can also evoke controversial emotions in people, such as fear, resentment, and confusion [12,13] which result from a high level of daily uncertainty. These emotions contribute to negative impacts on psychological wellbeing (PWB) [11,14,15]. 

In this context, Spain was one of the first affected countries. In the face of an increasing number of infected cases, the Spanish government decreed a “state of emergency” on 15 March, lasting until 21 June, which included a period of quarantine until 2 May. This measure also included the closure of educational and business centers, and non-essential factories and stores such as malls, restaurants, and coffee shops [16]. These measures led to conditions of social distancing and movement limitation [14]. Given this situation, a spontaneous migration from urban to rural environments took place among populations globally [17] in the search for better life conditions under the belief that less crowded regions would be safer [18] but also increasing the risk of spreading the virus more quickly [19].

During COVID-19 quarantine, most people faced an unknown situation and feared an indeterminate future, leading to feelings of stress, anxiety, and depression [20], related to difficulties in, for example, obtaining food or basic supplies [21,22]. However, these negative feelings may have a greater impact depending on an individual’s characteristics [14]. One of the most demanding characteristics of the quarantine is that it restricts people’s social relationships and imposes limits on their movement [23,24], which impacts directly on social determinants and, hence, on people’s behaviors [25]. Nevertheless, the current level of technological development offers the population not only suitable modes of communication, but also a preferable means of learning social rules, recreation and entertainment, transfer of experiences, friend-making, and dating [26,27]. Thus, once individuals’ basic needs have been met, quarantine can also provide people the opportunity to acquire new skills or healthy habits in their daily routines. That is, among the negative consequences of the quarantine, restrictions on social relationships and limits on movement may also provide the opportunity to manage time in different ways, which can lead, in some cases, to more efficient behaviors. Among these approaches to mitigating the negative effects of confinement, experts have encouraged the population to adopt healthy habits [23] based on the three pillars of health promotion and well-being: physical health, healthy eating, and emotional wellness [28]. 

It is currently considered that healthy habits are not limited to exercise and nutrition behaviors. Thus, Cognitive Fitness [29], a recent concept proposed by Aidman on the basis of Seligman’s Positive Health definition [30], enables the application of knowledge, skills, and attitudes in the development of task performance, which influences mental health [31] from a holistic and practical perspective that describes how these faculties are enhanced and decline across the real-life processes of maturation, ageing, training and education, medical treatment, social awareness and others. This research adopts this new concept of healthy habits, studying the extent to which people initiated such habits during quarantine. In addition, there is now little doubt about the beneficial relationship between healthy exercise routines, the prevention and treatment of diseases, and the enhancement of PWB [32,33,34]. In the case of the pandemic, the role of physical exercise—specifically, the best option to enhance physical activity (PA) levels—is even more crucial, because exercise may have a protective effect on the immune system and provide a proper response to the threat of the disease [35]. Similarly, eating habits are also a significant global concern [36] because they are considered a risk factor that causes more deaths than other factors such as tobacco consumption [37]. It has been shown that a diet with a high intake of fruits and vegetables, whole grain, fish, olive oil, and low-fat dairy antioxidants presents benefits for PWB [38]. Despite the fact that there is not a direct relationship between home meals and dietary quality, consuming home-cooked main meals has been associated more frequently with a healthier diet [39]. Based on the aforementioned issues, pandemic confinement may be considered a trigger to enhance healthy behaviors related to exercise, eating, and personal interests. 

Both physical exercise and diet have been related to health [40], but the relationship between them has also been studied. Unlike those who practice a diet, who are encouraged to focus just on decreasing their calorie intakes, those who begin to exercise also adopt healthy eating habits [41]. The present research evaluates, on the one hand, the intensity and type of PA, as well as the type of diet that people followed. On the other hand, it studies whether these people initiated healthy habits related to physical, nutritional, and emotional health during confinement.

As mentioned above, evidence exists of the negative effects of the outbreak of COVID-19 and the measures taken to control it (i.e., confinement, quarantine) on the PWB of people in the first countries to be severely affected, such as China [42,43] and Italy [44]. Studies have shown that the relationship between PWB and PA can be bidirectional [45,46]. This means that not only can PA relate to PWB, but levels of PWB can also increase PA levels. Since these two variables are strongly interrelated, the effect of COVID-19 quarantine on PWB may affect individual PA levels. PWB has also been linked to, among other factors, a reduced risk of mortality and cardiovascular disease [47,48]. These positive consequences may be due, in part, to healthy behaviors in which satisfied and determined individuals are more likely to participate. Conversely, there is also evidence that PWB assessed before measuring diet predicts subsequent fruit and vegetable consumption after taking into account a wide range of possible confounding factors, including depression [47], which suggests that increased PWB contributes to the adoption of healthy behaviors.

In summary, although social determinants are usually used to understand the social distribution of disease, its relationship to behavior changes is unknown [49]. There are many questions about how social determinants mediate or moderate the acquisition of new healthy habits. In this study, we argue that of quarantine affects many social determinants and people’s behaviors people. Thus, this research proposed the following objectives.

**Figure 1:**
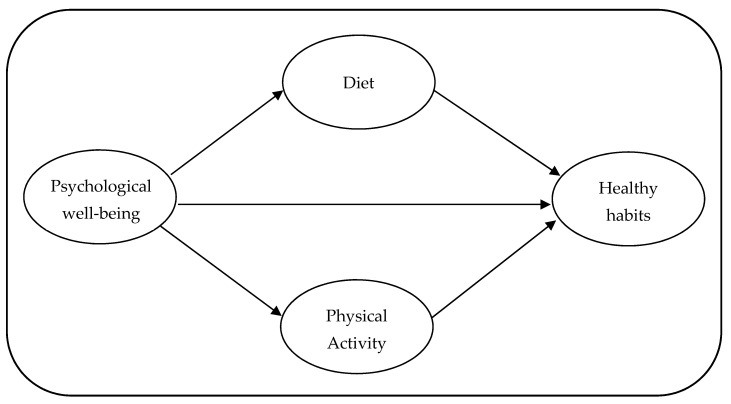
Model of relationships between PWB and healthy habits with the mediation of diet and PA.

- Objective 1: to verify if the PWB of people, derived from the quarantine situation, relates to healthy habits, and if PA and diet mediate this relationship (see Figure 1).
- Objective 2: to test if there are differences in this model of relationships between women and men.
- Objective 3: to analyze if there are differences in the levels of healthy habits, PA, diet, and PWB depending on gender.
- Objective 4: to test if there are differences in the levels of healthy habits, PA, diet, and PWB depending on living area.
- Objective 5: to assess if there are interaction effects of gender and living area in the levels of healthy habits, PA, diet, and PWB.

## 2. Materials and Methods

### 2.1. Participants

We established three inclusion criteria for participants: (1) to be living in Spain at the time of confinement; (2) to be at least 18 years old; and (3) to accept participation in the study in the online questionnaire. In addition, we established an exclusion criterion, that is, not having completed all the questions in the questionnaire (except for those that were not mandatory, such as political orientation). The study was conducted during the period from 26 April to 22 May 2020, during which 2216 people started the questionnaire. Of these, only 1509 met the inclusion and exclusion criteria, and therefore constitute the research sample (1020 women, 67.6%). 

The average age of the participants was 37.23 years old (SD = 13.91), ranging from 18 to 78. The main occupations of people were: 32.8% worked online, 25.6% were students, 12.1% were in a temporary employment regulation file (ERTE in Spanish), 11.5% were unemployed, and 9.7% worked face-to-face. Moreover, 29.5% had, at least, one child. The average period of confinement was 45 days (SD = 3) ranging from 33 to 68. Only 6.8% of the sample had isolated due to COVID-19. 

### 2.2. Materials

For data collection, a questionnaire composed of several scales was created. The variables that were evaluated were the following:

(a) Demographic variables such as gender, age, country in which people live during confinement, work occupation, living area in which people live during confinement (i.e., urban area or rural area), number of children, political orientation, professed religion, pets, or time of confinement. 

(b) To assess the number of healthy habits performed during confinement, we created an ad hoc questionnaire consisting of 17 items that were answered according to a scale of two alternatives comprising 0 (“No”) and 1 (“Yes”). The questionnaire began with the following sentence: “During confinement, what kind of habit have you started?” The 17 items were classified in the following three categories according to the three pillars of health promotion and well-being [28]: (1) habits related to physical health, with 7 items (e.g., “I have done more physical exercise”, “I have smoked less”); (2) habits related to nutritional health, with 3 items (e.g., “I have used less salt to eat”, “I have done intermittent fasting”); and (3) habits related to emotional health, with 8 items (e.g., “I have practiced mindfulness”, “I have practiced meditation”). The maximum score that could be obtained was the result of the sum of all habits, that is, 17 points, while the minimum score was 0 points. For the creation of the items that composed the questionnaire, we collaborated with two experts in health promotion, who verified that the items made it possible to evaluate the mentioned pillars of health promotion and well-being. The items were created following the guidelines established by Alaminos and Castejón [50]: each item should address only a single issue; items should be clear, simple, and concise; items with repeated information should be avoided; the vocabulary should be accessible to all members of the population; and biased items should be avoided. For this purpose, in a first phase each expert individually proposed several healthy habits. In a second phase, each expert reviewed the proposals of the other expert. Finally, both experts met to select the final items. 

(c) To estimate the level of PA, we used the official short form version of the International Physical Activity Questionnaire (IPAQ) [51], validated in Spanish [52]. The IPAQ questionnaire consists of seven generic items. This measure assesses the types of intensity of PA that people conduct as part of their daily lives, which are considered to estimate total PA in MET-min/week and time spent sitting. IPAQ defines three categories of PA: “low” (physically inactive), “moderate”, and “high” (vigorous) levels of PA, in relation to health-related recommendations [53]. All items refer to the activities during the previous seven days. IPAQ has acceptable measurement properties (e.g., test–retest = 0.76) for monitoring population levels of PA among 18-to 75-year-old adults in diverse settings [51]

(d) To measure PWB we used the 12-item version of the General Health Questionnaire (GHQ-12) [54], validated in Spanish [55]. The questionnaire assesses psychological health problems over the past few weeks using a 4-point scale. Specifically, each item is accompanied by four possible responses, typically being “not at all”, “no more than usual”, “rather more than usual”, and “much more than usual”, scoring from 1 to 4, respectively. An example of an item is “Have you recently been feeling unhappy and depressed?”, and high scores indicate better psychological well-being. According to previous research [56,57], the items can be grouped into three dimensions: (1) successful coping, which includes 6 items (e.g., “Have you recently been able to concentrate on whatever you are doing?”; (2) self-esteem, which includes 3 items (e.g., “Have you recently been feeling reasonably happy, all things considered?”); and (3) stress, which includes 3 items (e.g., “Have you ever felt that you can’t overcome your difficulties?”). The questionnaire has shown good reliability alphas indices in different studies, ranging from 0.76 [58] to 0.90 [59].

(e) To obtain information about the type of diet of the participants, we created a questionnaire based on the healthy eating pyramid for healthy adults proposed by the Spanish Society of Community Nutrition (SENC in Spanish) and the Spanish Society of Family and Community Medicine (SemFYC in Spanish) [60]. For the creation of the items that comprised the questionnaire we collaborated with three experts in nutrition, who verified that the items made it possible to evaluate the mentioned healthy eating pyramid. As in the case of the healthy habits questionnaire, the items were created following the guidelines established by Alaminos and Castejón [50]. The final questionnaire was composed of 13 items that were answered according to a scale of four alternatives, which was adjusted to the item being asked. An example of an item is “During confinement, how many times do you usually eat fish and shellfish?” In this example the response scale is “1: Never or occasionally”, “2 = 1–2 times per week”, “3 = 3–4 times per week” and “4 = More than 4 times per week”. The final scores for each item were recoded according to the recommendations of the SENC and the SemFYC [60], differentiating three levels of suitability: harmful, improvable, and recommended. In the case of the example, final scores appeared as: “Never or occasionally = 0” (because it is harmful to eat so little each week), “1–2 times per week = 1” (because it is not recommended to eat so little), “3–4 times per week = 2” (because these are the recommended amounts), and “More than 4 times per week = 1” (because it is not recommended to eat so much). The 13 items were classified into 3 categories according to an exploratory factor analysis computed with SPSS: (1) plant-based food, which includes 5 items (e.g.,“…vegetables”); (2) meat-based food, which includes 5 items (e.g.,“…eggs”); and (3) processed food, which includes 3 items (e.g., “… sweets and/or sodas“).

### 2.3. Design and Procedure

A quantitative research approach with a cross-sectional design was adopted in this study. The online software provided by www.onlineencuesta.es (accessed on 1 April 2020) was used to create and distribute the questionnaire. The questionnaires were administered online between 26 April and 22 May 2020 and disseminated through various news websites and social networks such as LinkedIn, which reported the start of research into the effects of confinement on people’s well-being.

The first page of the questionnaire contained information on the anonymity and confidentiality of the responses, as well as a request for consent for processing. In addition, information was provided on the purpose of the research, the persons targeted, the duration of the questionnaire, and the data protection regulations on which it was based.

The time required to answer the questionnaire was approximately 15 min. The participants were able to answer the questionnaire at different times, recording a code that the questionnaire showed them on the upper left of the screen. The responses to the questionnaires were stored in an online database to which the authors had access at the end of the questionnaire administration period to download the data and proceed with their analysis.

### 2.4. Data Analysis

All analyses were conducted with the SPSS Statistical Package (Version 25, IBM Corp, Armonk, NY, USA), and the level of significance was set at *p* ≤ 0.05. First, a series of χ^2^ were conducted to test any potential effects of participants’ demographics in gender differences. 

Second, descriptive analyses and intercorrelations among dependent variables were computed. Third, to test the relationship among PWB, diet, PA, and healthy habits, Structural Equation Models (SEM) were calculated using AMOS software (Version 25, IBM Corp, Armonk, NY, USA). Each of the model’s latent variables incorporated three different indicators. In the case of PWB, the items were grouped according to the three dimensions that differentiate the questionnaire: (1) successful coping; (2) self-esteem; and (3) stress. The items of PA were also grouped according to the dimensions of the questionnaire: (1) vigorous PA; (2) moderate PA; and (3) low PA. Diet items were grouped according to the type of food they referred to: (1) plant-based, (2) meat-based, and (3) processed foods. Finally, healthy habits were grouped according to the three pillars of health and wellness promotion in (1) physical health; (2) nutritional health; and (3) emotional health. All factors loadings were significant. Fourth, to explore possible differences in results between genders, MuLti-Group (MLG) analyses were performed. This technique looks for statistically significant differences in trajectory coefficients between sub-samples [61]. Finally, to analyze differences in healthy habits, diet, PA, and PWB, several 2 (gender) × 2 (living area) MANCOVAs were conducted. Work situation and political orientation were considered covariables. Effect sizes (ŋ^2partial^) for main effects and interactions were included. The minimum sample needed to be able to apply a 2 × 2 MANCOVA with these variables according to the G*Power3 program [62] (effect size = 0.06; power = 0.95) is 186. Therefore, the research sample is suitable to compute this type of analysis.

We used maximum likelihood estimation methods, and the input for each analysis was the covariance matrix of the variables. We tested different fit indices: the χ^2^ Goodness-of-Fit Statistic, Goodness-of-Fit Index (GFI), Adjusted Goodness-of-Fit Index (AGFI), Root Mean Square Error of Approximation (RMSEA), Tucker–Lewis Index (TLI), Comparative Fit Index (CFI), the Normed Fit Index (NFI), and the Incremental Fit Index (IFI). According to Browne and Cudeck [63], values smaller than 0.05 for RMSEA indicate a good fit and values between 0.05 and 0.08 indicate an acceptable fit. For the remaining indices, values greater than 0.90 indicate a good fit. A revised cut-off value of CFI close to 0.95 is also advised [64].

## 3. Results

### 3.1. Reliability Analysis

Reliability as an internal consistency was calculated through Cronbach’s alpha only for the PWB scale and its dimensions, since diet, PA, and healthy habits items are not susceptible to misinterpretation. The minimum value considered adequate is 0.70 [65], which is appropriated for PWB (α = 0.89), for successful coping (α = 0.75), and for self-esteem (α = 0.77). The value of the stress dimension was close to 0.70 (α = 0.63).

### 3.2. Descriptive Analysis

On the one hand, several χ^2^ were conducted to examine any potential effects of participants’ demographics (i.e., work situation, number of children, and professed religion) in gender differences. Adjusted residuals were included in the χ^2^ test when one of the variables distinguished more than two categories to test among the categories in which there were differences (adjusted residual ≥ 1.96). Significant differences between women and men were found between count and expected count depending on work situation (χ^2^(5) = 22.31, *p* = 0.001, see Table 1). Specifically, there were fewer women than expected working online and face-to-face, and more men than expected. No significant differences were found depending on the number of children (χ^2^(3) = 3.79, *p* = 0.28) and professed religion (χ^2^(3) = 6.02, *p* = 0.11).

On the other hand, as can be seen in Table 2, healthy habits are positively and significantly related to diet and PA. PA is also significantly and positively related to diet and PWB. Finally, diet and PWB are positively and significantly related. There is no statistically significant relationship between PWB and healthy habits, which will be discussed further in the following section on inferential analyses.

### 3.3. SEM and MLG Analyses

The common method variance for the variables was tested using Harman’s single factor test with CFA (e.g., [66]), which revealed that a single factor could not account for the variance in the data (χ^2^ (54) = 718.66, *p* < 0.0001). Consequently, the common method variance is not a deficiency in the dataset.

**Figure 2:**
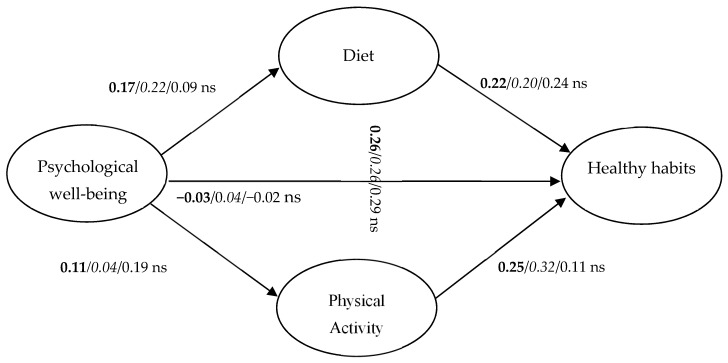
Model objective 1 and objective 2 (**both**/*women*/men); ns = non-significant (*p* > 0.05).

To contrast objective 1, which is intended to verify if the PWB of people relates to healthy habits, and if diet and PA mediate this relationship, an SEM was performed. The model fits the data well (χ^2^ = 132.23, df = 48, GFI = 0.99, AGFI = 0.98, RMSEA = 0.03, TLI = 0.97, CFI = 0.98, NFI = 0.96, IFI = 0.98). As can be seen in Figure 2, PWB is not related to healthy habits (*p* > 0.05). This relationship is fully mediated by diet (which is positively related to PWB and healthy habits) and PA (which is also associated significantly and positively with PWB and healthy habits).

To contrast objective 2, which is intended to test if there are differences in the model of relationships between women and men, an MLG was performed. The model fits the data well (χ^2^ = 188.9, df = 96, GFI = 0.98, AGFI = 0.97, RMSEA = 0.02, TLI = 0.96, CFI = 0.97, NFI = 0.95, IFI = 0.97). PWB is not directly related to healthy habits in either women or men. Diet fully mediates the relationship in women, but not in men. PA does not mediate the relationship between PWB and healthy habits in either gender. In men, PWB is associated with PA, PA with diet, and diet with healthy habits (see Figure 2).

### 3.4. Gender Comparisons

To contrast objective 3, which is intended to analyze if there are differences in the levels of healthy habits, PA, diet, and PWB depending on gender, a MANCOVA was conducted. There was an overall effect of gender on dependent variables (F(4, 1431) = 9.06, *p* < 0.0001, ŋ^2partial^ = 0.025). Moreover, as can be seen in Table 3, women developed more healthy habits than men. On the other hand, men scored significantly higher in PA and PWB than women. No gender differences were found in diet. 

### 3.5. Living Area Comparisons

To contrast objective 4, which is intended to test if there are differences in the levels of healthy habits, PA, diet, and PWB depending on living area, a MANCOVA was conducted. There was an overall effect of living area on dependent variables (F(4, 1431) = 3.94, *p* < 0.003, ŋ^2partial^ = 0.011). Moreover, as can be seen in Table 4, people who lived in an urban area showed lower levels of PA and higher levels of PWB, compared to those who lived in a rural area. No significant differences were found in relation to healthy habits and diet.

### 3.6. Interactions between Gender and Living Area

To contrast objective 5, which is intended to assess if there are interaction effects of gender and living area on the levels of healthy habits, PA, diet, and PWB, a MANCOVA was conducted. No overall effect was found (F(4, 1431) = 0.96, *p* = 0.43, ŋ^2partial^ = 0.003) nor any interaction effect of the independent variables in healthy habits (F(1, 1434) = 1.94, *p* = 0.16, ŋ^2partial^ = 0.001), PA (F(1, 1434) = 1.34, *p* = 0.25, ŋ^2partial^ = 0.0001), diet (F(1, 1434) = 0.003, *p* = 0.96, ŋ^2partial^ = 0.0001), or PWB (F(1, 1434) = 0.103, *p* = 0.75, ŋ^2partial^ = 0.0001). 

## 4. Discussion

This study aimed to analyze how PWB relates to healthy habits during confinement and whether there was any difference by gender and by living area, thus establishing a relationship model between PWB and the development of healthy habits, mediated by diet and PA. 

Our findings showed that the relationship between PWB and healthy habits was fully mediated in a positive way by diet and by PA (objective 1). This result provides evidence that, when people report good levels of PWB and have a good perception of their health, they may not feel the need to develop new healthy habits. Only those who decided to have a balanced diet or practice PA also considered increasing their healthy habits [67,68] during quarantine. This result also provides evidence that having a healthy diet is related to those action-oriented individuals who also tend to enroll in other healthy plans [69,70].

When we tested the model on women and men separately (objective 2), we found that the full mediation of diet between PWB and healthy habits existed in women, but not in men. Interestingly, in men the high levels of PWB were related to the participation in more PA. This was related to the development of a more balanced diet, which was associated with developing heathier habits. As noted above, this result is consistent with other research showing that it is common for people who are physically active to also be concerned about having a balanced diet [41]. Our findings can be conditioned by the idea that health is defined by the physical, social, cultural, and economic environment in which people live and work [71]. Therefore, we can suggest that when people are concerned about health, they harbor this concern for all facets of their lives. 

Previous studies have shown that men are generally more physically active than women [72], while women tend to follow a healthier diet [73,74]. The results of our study (objective 3) are partially consistent with these previous findings. In terms of PA, previous studies suggest that the main reason for exercising during confinement was people’s pre-existing habits. Therefore, the more physical exercise was performed prior to confinement, the more physical exercise was done during confinement [75]. Thus, despite changes towards gender equality in many fields [76] and narrowing of the gender gap [77,78], it appears that gender stereotypes were displayed in PA levels and diet during confinement. More studies are needed to determine if the differences between men’s and women’s diet disappeared, as has been found in previous research [79]. 

Regarding the other dependent variables in which there were also differences, our results showed that women developed more healthy habits, but they experienced lower levels of PWB. The impact of the restriction on social relationships and the limitation of movements on social determinants [23,80] suggests that women developed more healthy habits than men. Previous studies have found that the greater number of healthy activities in women was related to an increase in their perceived stress and other health problems such as headaches [81], which is consistent with our results concerning levels of PWB. Although gender inequalities are more significant in developing countries [82], numerous inequalities also exist in richer countries, and relate mainly to socioeconomic and psychosocial circumstances. It is now well known that health and quality of life are a social result directly related to people’s general life conditions and lifestyles [83], and that these conditions can be severely affected by the type of isolation experienced as a circumstance of the confinement.

In addition to examining gender differences, we were also interested in studying the effect of living areas on healthy habits, PA, diet, and PWB (objective 4). Our results suggest that people who lived in rural areas were physically more active than those who lived in urban areas. As differences in healthy diets and habits were not significant between the two environments, we suggest that although lifestyles in rural areas were more active than those in urban areas, this is not due to healthy physical exercise routines but to the PA required by the environment in which people live. These findings are in accordance with a wide selection of the literature that suggests that people who live in rural areas develop less healthy habits of physical exercise than those who live in urban areas [84,85]. 

Nonetheless, previous research suggests that rural areas were related to positive levels of PWB [86], whereas other studies highlighted the negative effect of isolation caused by living in less densely populated areas [87]. Our findings were in line with this last insight, since they showed that people living in rural areas had lower levels of PWB than those living in urban areas. A country’s development level allows for a better understanding of this outcome. Although people claim to be happier in rural areas in less developed countries, in more developed countries urban areas show better levels of life satisfaction [88]. Therefore, our results provide evidence that the facilities and services provided by urban areas are more relevant to maintaining a good level of PWB than other aspects, such as lower population density or access to nature, which are characteristics of rural areas, including during confinement.

Finally, we wished to determine if there was an interaction between gender and living area (objective 5). In this sense, no interaction effects were found in any of the dependent variables. Although the difference between men and women was greater in the number of healthy habits developed in rural areas (women developed more) compared to urban areas, the interaction effect was not large enough to be significant. Therefore, the differences found according to gender or the area in which people lived remained unaltered when assessed together. 

One limitation of this study is that the results were obtained by self-reported measures, and consequently may be contaminated by the common method variance and by the wish to answer consistently [89]. In order to control for this, we checked the potential impact of the common method variance in our data [90]. Although we cannot completely rule out the possibility that the common method variance bias played a role, our tests indicated it was not present.

Furthermore, we assumed a unidirectional view of the relationships among the variables measured, although these could be bidirectional. As the relationships between the variables studied could also be explained in the opposite direction (that is, that healthy habits, healthy diets, or PA are associated with people’s PWB [68,91]) in future research it would be useful to develop longitudinal designs instead of cross-sectional designs to uncover reciprocal causal relationships.

It should also be noted that a portion of the participants’ responses were obtained after quarantine, which could have affected the results. However, this is very unlikely because most of the responses (87%) were received during quarantine and, in addition, no differences were obtained in the variables analyzed when comparing the responses given before and after that period. 

Finally, we are aware of the exceptional circumstances of the COVID-19 quarantine, which was the first situation of its kind faced in modern history. Thus, both the behavior of the population and the measures taken by governments and health services could be different in similar scenarios.

## 5. Conclusions

Have we taken advantage of the quarantine to develop healthy habits? The answer is yes, but some clarifications are needed. In general, those people who experienced better levels of PWB during quarantine developed more healthy habits than those who experienced lower levels of PWB. However, to increase their number of healthy habits, they also had to improve their diet (especially women) and their PA (especially men), as no direct relationship was found between PWB and the development of healthy habits. Although isolation measures do affect people’s PWB, this does not seem to be a barrier to the development of healthy habits during quarantine. In fact, despite social distance, the development of modern technology allowed people to maintain their healthy routines and even initiate new ones.

Did gender or the area in which people lived differentially affect the development of healthy habits, diet, PA, or PWB during quarantine? The answer is mixed across the variables examined: women showed more healthy habits and less PA and PWB than men; in contrast, in rural areas people showed more PA and less PWB than in urban areas.

In summary, the present study shows how Spanish people behaved during confinement in terms of the acquisition of healthy habits depending on gender and living area. The results should be taken into consideration in the planning of successful health promotion strategies in similar situations in the future, as it may be a profitable time to initiate valuable new habits. The findings encourage health care actors to take advantage of such situations to empower and engage people in healthy habits, especially but not limited to, those related to diet for women and PA for men.

## Figures and Tables

**Table 1:** Count, expected count, and adjusted residual of chi square analysis between gender and work situation.

		Women	Men
Unemployed	Count	127	46
	Expected count (%)	116.9 (12.5)	56.1 (9.4)
	Adjusted residual	1.7	−1.7
ERTE	Count	127	55
	Expected count (%)	122.9 (12.5)	59.1 (11.2)
	Adjusted residual	0.7	−0.7
Face-to-face work	Count	89	57
	Expected count (%)	98.6 (8.7)	47.4 (11.7)
	Adjusted residual	−18	1.8
Online work	Count	333	162
	Expected count (%)	334.4 (22.1)	160.6 (10.7)
	Adjusted residual	−0.2	0.2
Face-to-face and online work	Count	40	43
	Expected count (%)	56.1 (2.7)	26.9 (2.9)
	Adjusted residual	−3.9	3.9
Study	Count	274	112
	Expected count (%)	260.7 (18.2)	125.3 (7.4)
	Adjusted residual	1.7	−1.7
Another situation	Count	28	14
	Expected count (%)	28.4 (1.9)	13.6 (0.9)
	Adjusted residual	−0.1	0.1

ERTE (in Spanish): temporary employment regulation file.

**Table 2:** Means (M), Standard Deviations (SD), and correlations.

Variable	M ± SD	Correlations			
		(1)	(2)	(3)	(4)
1. Healthy habits	2.36 ± 1.67	1	0.12 **	0.13 **	0.02
2. PA	1864.80 ± 2918.31	-	1	0.13 **	0.08 **
3. Diet	10.02 ± 5.51	-	-	1	0.14 **
4. PWB	2.84 ± 5.79	-	-	-	1

Note. PA: Physical Activity; PWB: Psychological well-being; ** *p* ≤ 0.01.

**Table 3:** Descriptive statistics of healthy habits, PA, diet, and PWB by gender.

Variables	Women		Men		*F*	*p*	ŋ^2partial^
	n (%)	M ± SD	n (%)	M ± SD			
Healthy habits	974 (67.6)	2.51 ± 1.7	466 (32.4)	2.1 ± 1.61	15.26	0.0001	0.011
PA	974 (67.6)	1758.58 ± 2365.02	466 (32.4)	2038.23 ± 2753.05	4.75	0.03	0.003
Diet	974 (67.6)	10.12 ± 5.16	466 (32.4)	9.7 ± 5.16	0.76	0.38	0.001
PWB	974 (67.6)	2.78 ± 0.59	466 (32.4)	2.95 ± 0.53	12.06	0.001	0.008

Note. M: Mean; SD: Standard Deviation; PA: Physical Activity; PWB: Psychological well-being.

**Table 4:** Descriptive statistics of healthy habits, PA, diet, and PWB by living area.

Variables	Urban Area		Rural Area		*F*	*p*	ŋ^2partial^
	n (%)	M ± SD	n (%)	M ± SD			
Healthy habits	1222 (84.8)	2.38 ± 1.7	218 (15.2)	2.37 ± 1.68	0.90	0.34	0.001
PA	1222 (84.8)	1798.22 ± 2437.58	218 (15.2)	2134.18 ± 2811.65	4.91	0.03	0.003
Diet	1222 (84.8)	10.01 ± 5.19	218 (15.2)	9.81 ± 5.04	0.26	0.61	0.0001
PWB	1222 (84.8)	2.86 ± 0.57	218 (15.2)	2.7 ± 0.59	8.61	0.003	0.006

Note. M: Mean; SD: Standard Deviation; PA: Physical Activity; PWB: Psychological well-being.

## Data Availability

The datasets generated and/or analyzed during the current study are not publicly available but are available from the corresponding author on reasonable request.

## Abbreviations

GHQ	General Health Questionnaire
IPAQ	International Physical Activity Questionnaire
MLG	Multi-Group
PA	Physical Activity
PWB	Psychological wellbeing
SEM	Structural Equation Models
WHO	World Health Organization
